# Supplementary material for: Australian Culex annulirostris mosquitoes are competent vectors for Japanese encephalitis virus genotype IV
Source: Emerg Microbes Infect. 2024 Nov 12;13(1):2429628. doi: 10.1080/22221751.2024.2429628 (PMC11587722; doi:10.1080/22221751.2024.2429628)

**Supplementary Data:**

**Supplemetary Figure 1.** To determine the absolute number of genomic copies of JEV and 18s gene in each mosquito sample, synthetic JEV NSA2 or *Cx. annulirostris* 18s ribosomal RNA was designed based on target sequences and used to generate standard curves. Serial 10- fold dilutions of RNA were prepared in Tris-EDTA buffer and each dilution was tested via RT-qPCR. The limit of detection was 120 JE viral genomic copies and 12 genomic copies for 18s. Linear equations were derived from the standard curves, enabling normalized JE viral genomic copy numbers to be calculated from C_T_ values obtained from RT- qPCR testing of JEV-infected samples.

*JEV NSA2 synthetic RNA sequence:*

GCCACCCAGGAGGTCCTTCGCAAGAGGTGGACGGCCAGATTGACAGTTCCTGCGGTTTTGGG

*Standard Curve for JEV*


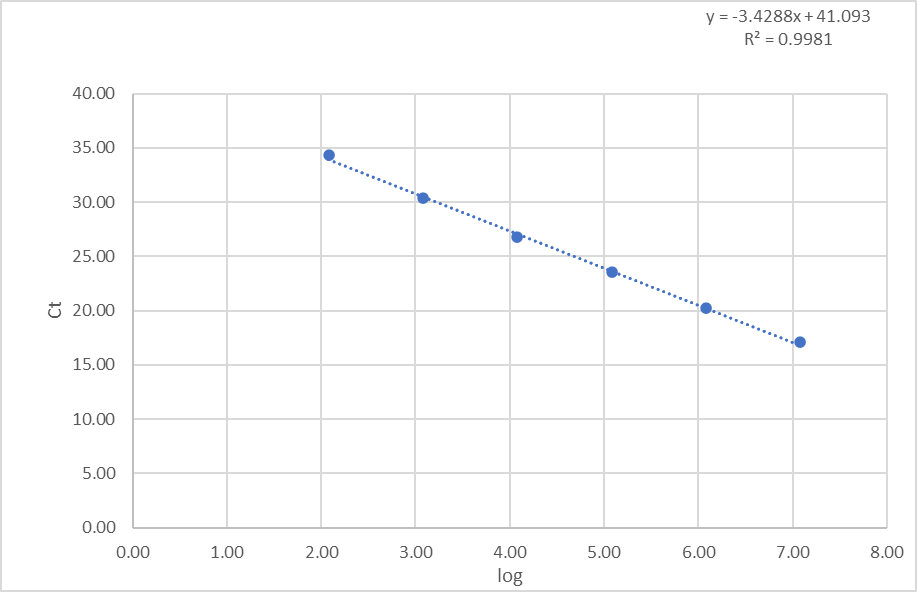


*Cx. Annulirostris 18S synthetic RNA sequence:*

CACGTGCGTACGGTAGAGAGACAGAGAGAAAACCCTAGGCTGGTCAGGTCCGGATCGCGATACCGAGCGAGCGCGGTCGGCAGGTTCGTCCTATGTCCGACACACC

*Standard Curve for 18s*


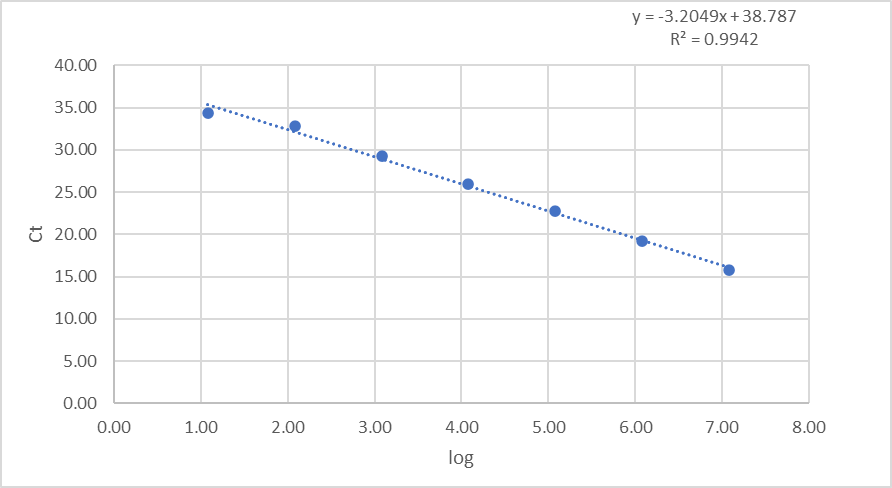


**Supplementary Figure 2**: Statistical analysis was performed using R version 4.4.0 for formal confirmatory analyses undertaken to determine the effect of the viral strain and the mosquito samples on the log10 mean copy number per mg of tissue or saliva. The experimental design was treated as factorial and analysed using a two-way Analysis of Variance (ANOVA) implemented using the “*lm*” function from the core R *stats* library. The assumptions of the linear model were assessed visually through examination of the residuals of the ordinary least square (OLS) fitted model, which demonstrated a high degree of unequal variance (heteroscedasticity), due mainly to the high variability of the saliva sampling. To mitigate the impact of this on the accuracy of the parameter values, we used a weighted least squares (WLS) regression approach, with the weighting calculated as the inverse of the squared fitted values from the initial OLS regression. Following detection of significant overall differences between group means (p <0.05), post hoc testing to define the significantly different groups was undertaken using the “emmeans” and “multcomp*”* R packages.


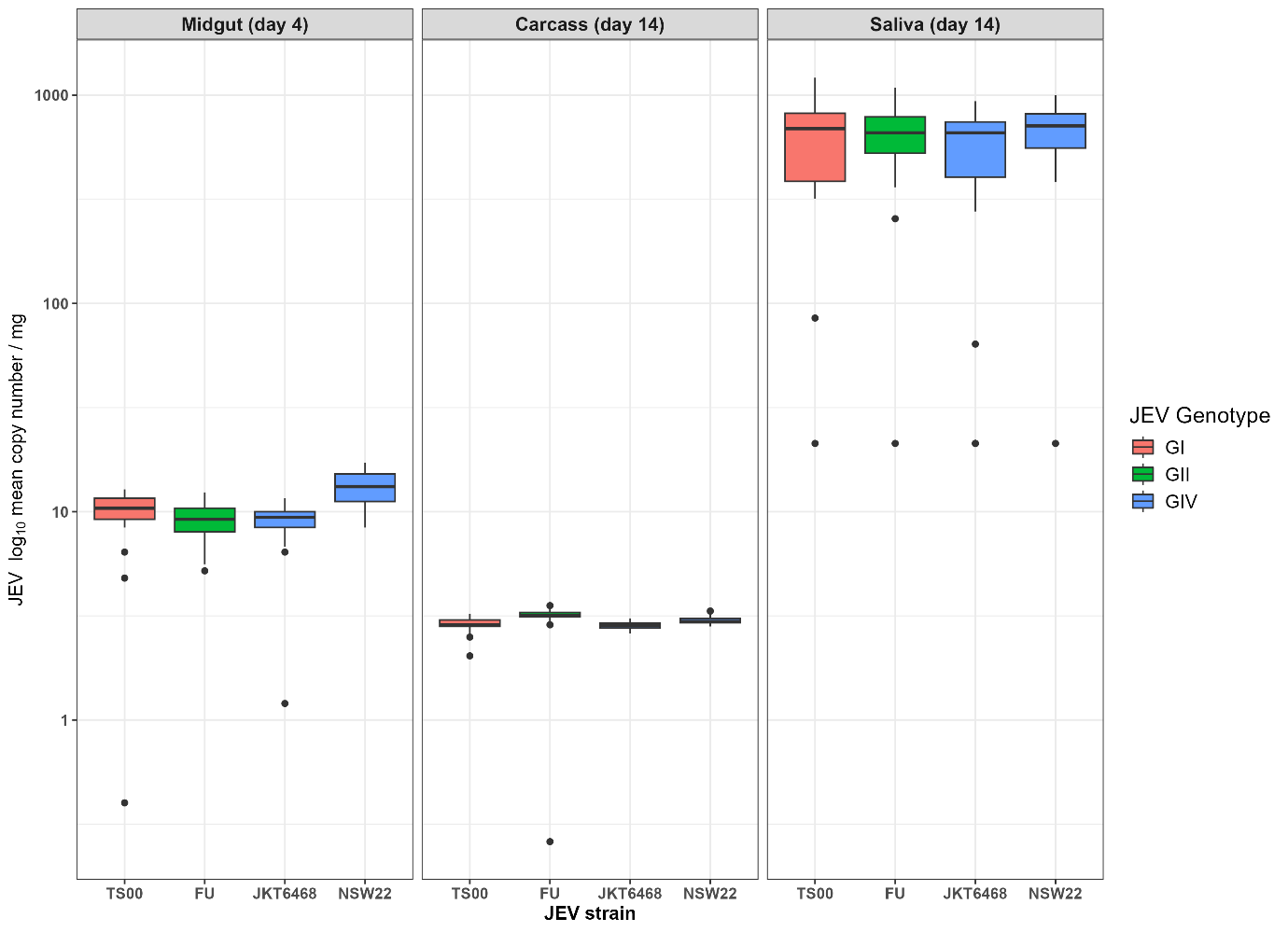

Supplement: SuppData.docx [file TEMI_A_2429628_SM7640.docx]
